# The Promotion of Sustainable Diets in the Healthcare System and Implications for Health Professionals: A Scoping Review

**DOI:** 10.3390/nu13030747

**Published:** 2021-02-26

**Authors:** Goiuri Alberdi, Mirene Begiristain-Zubillaga

**Affiliations:** Department of Finance Economy II, Faculty of Economics and Business, University of the Basque Country (UPV-EHU), Plaza de Oñati 1, 20018 Donostia-San Sebastián, Basque Country, Spain; goiuri.alberdi@ehu.eus

**Keywords:** sustainable diet promotion, healthcare system, food–health environments, dietitian, health professionals

## Abstract

The impacts of the current global food system are already visible in the environment and in the health of the population. The promotion of sustainable diets is key to counter the negative consequences. The healthcare system could be a powerful tool to educate patients by guiding their diets towards sustainability. This study aimed to assess the size and scope of the available literature regarding the promotion of sustainable diets in the healthcare system and to obtain a reliable approximation of the processes and roles related to sustainable diet promotion within healthcare systems. A scoping review where online databases were used to identify English written scientific and grey literature published between 2000–2019 was carried out. The analytical–synthetic approach was used for data charting. Twelve studies were included that were published between 2007–2020. The data highlight education, community and clinical health services, community engagement and policy advocacy, and governance as main action areas along with two transversal aspects, social support, and gender. A systemic approach to the food system is emphasized. Evidence suggests that health professionals have the potential to drive a paradigm shift in food–health environments. Currently, however, their role and potential impact is underestimated within healthcare systems. This review has identified a framework with key areas where processes need to be developed to guarantee sustainable diet promotion in healthcare services.

## 1. Introduction

Food is a central and essential component of our lives. The food that we produce, distribute, consume and waste is already having major impacts on environment degradation, climate change and the health of the population [1,2,3]. Food systems contribute up to 26% of greenhouse gas emissions (GHGE), where the rearing of intensive livestock production models is by far the biggest contributor [4,5,6,7]. Likewise, conventional agriculture practices are also leading to land degradation, with soil losses affecting up to more than 11% of the territory in the European Union (EU) [8,9].

The use of pesticides and nitrogen-based fertilizers leads to impacts on plant and insect life, and the resulting biodiversity loss jeopardizes a range of environmental services, including the pollination of many food crops, threatening future yields and costing about 3% of the global Gross Domestic Product (GDP) yearly [9].

In addition to issues related to production, the food system has been globalized over recent decades due to, among other things, the evolution of refrigerated transport, communication technology and reduced trade barriers [10]. Consequently, consumer decisions (especially in urban areas) are made in disconnect from the environmental and social impacts that are incurred elsewhere when producing food [11]. Globalization of food trade in agricultural products goes against the key concepts of sustainable agriculture, particularly local supply on a smaller scale and marketing systems that reduce reliance on off-farm resources and nourish local ecosystems and economies [11]. In addition, this disconnection, and its economic and social impacts, favor the consumption of processed and ultra-processed foods, and the impossibility of democratically accessing a healthy diet [12].

Dietary patterns are shifting, with more food of animal origin being consumed globally [3,13]. If this trend continues, global demand for meat and animal-based food products is likely to increase by 95%, which will boost food-related GHGE from 30% to 80% by 2050 [14]. These unsustainable practices, along with the growing competition for land, water and energy, will intensify in an expanding global population, affecting the capacity for future sustainable healthy food production [3].

On the other hand, food waste is a global issue due to its environmental, economic, and social impacts, requiring a change in political actions [15]. Twenty percent of the food produced in the EU is wasted, costing 143 billion euros annually [9]. This social and ethical dilemma touches upon the millions of undernourished people, while excessive consumption in high-income countries leads to billions of tonnes of quality food thrown away [15].

This excessive consumption has led to epidemic proportions of diet-related non-communicable chronic diseases, where currently seven of the top ten causes of death are linked to diet [3]. Young children are developing diseases rarely seen in childhood, such as type II diabetes, to the point of predicting that the children of today will have a shorter lifespan than their parents [16].

These series of negative externalities of the current food systems, from production to consumption, are not reflected in the cost of food, resulting in the need for the public sector to absorb these expensive costs in healthcare and in ecosystems [17,18]. Moreover, data from 2015 shows that diet-related diseases comprised 7.8% of the GDP of public expenditure on health in the EU, and it is thus considered among the largest and fastest growing spending item for governments [19].

Altogether, these data show that current global food systems are unsustainable and non-resilient, and this has been further exacerbated during the Coronavirus Disease (COVID-19) outbreak [20]. Even though our food systems are part of the problem, they can also be part of the solution. However, healthy diets alone do not produce substantial positive environmental effect (i.e., reductions in GHGE); hence, dietary guidelines need to include recommendations for environmental sustainability [21]. In that sense, more voices are defending the need for a social paradigm change, including the expansion of food citizenship, aligning health and environmental goals in the process, reducing the economic costs of unsustainability and ensuring a more resilient and localized food system [22,23,24].

The United Nations Decade of Action on Nutrition (2016–2025) is positioned to use the “opportunity to transform our current food system into one that is sustainable, resilient and provides healthy diets for all” [25]. In addition, sustainable food systems directly and indirectly influence the framework of the United Nations’ Sustainable Development Goals (SDG) [26,27]. Both agendas reflect the importance of education as a central criterion in the construction of sustainable food systems.

Specifically, the wider educational role of hospitals is being acknowledged, with attempts to improve the diets of patients even after they leave a care facility [28]. Health professionals are widely recognized experts who bring tremendous credibility and influence. However, as Mozaffarian et al. indicate, one of the least used settings to promote better nutrition is paradoxically the healthcare system [29]. The need to switch from a classical nutrient-adequate diet into a sustainable healthy diet is essential, but policies are still undeveloped in that area within the health services. Dietitians and health professional allies have a fundamental role to play in supporting people in becoming food citizens by educating and promoting the consumption of sustainable diets.

Encouraging sustainable diet intake and reducing food waste are part of the main solutions to reduce environmental detriment and to improve the health of the population [8,30,31,32]. Applying the sustainability criteria implies understanding the linkages between diets, agricultural production practices, environmental degradation and social impacts, including health, economy and governance of our food systems [33]. This is linked to the implementation and rooting within a community of an appropriate sustainable food environment (SFE). An SFE is a human-built social environment that includes the physical, social, economic, cultural and political factors that affect the accessibility, availability and adequacy of food within a community [34].

Specific scientific literature on the potential role of health professionals in driving the sustainability agenda within the clinical and community settings of healthcare services is relatively scarce. This paper aims to conduct a scoping review in order to obtain a preliminary assessment of the potential size and scope of the available literature regarding the promotion of sustainable diets in the healthcare system, and to obtain a reliable approximation of the current situation of the role of health professionals in sustainable diet promotion within healthcare systems. The results will shed light on gaps and new initiatives that are required to normalize the implementation of sustainable diet promotion in hospitals.

To achieve this objective, we first identify the key concepts related to the aims of this study. Next, we describe the methodological plan and protocol developed to carry out the scoping review study and the theoretical framework. With this framework, we present in detail the results obtained and discuss them by systematizing the content on key issues related to the study objective. Finally, we present the conclusions.

## 2. Materials and Methods

This type of literature review provides a preliminary assessment of the nature, potential size and scope of the research to date on the promotion of sustainable diets in the healthcare system [35].

The scoping review was conducted following the protocol explained by Arksey and O’Malley, the PRISMA extension for Scoping Reviews and others [36,37,38,39,40]. The protocol (available on request from the corresponding author) includes five steps: (1) define the research question; (2) identify relevant studies through a search strategy; (3) select studies following certain criteria; (4) data charting (extract, synthesize and interpret data); and (5) summarize and report the results.

The objective of the present review is addressed by the following research questions: what are the identified processes that are related with diet promotion/education in the European health service? How do they relate to sustainability dimensions (economic, social, environmental and cultural)?

### 2.1. Search Strategy

A three-step search strategy was developed to identify relevant studies. Firstly, related keywords (Table 1) derived from the research question were used for an extensive search of electronic databases including Web of Science, Proquest, Cochrane Library, Scopus, Emerald Insight, Pubmed/Medline, Dimensions, and Ovid. The searches were conducted in September 2019. Results were limited to documents written in English that were published between 2000 and 2019. Secondly, grey literature was obtained by hand-searching reports from relevant institutional websites (European Commission´s General Directorates; relevant organizations linked to food and health policymaking (such as Food and Agriculture Organization (FAO), World Health Organization (WHO) and NoHarm-EU), policy databases such as Nourishing and the global database on the Implementation of Nutrition Action). Additionally, the search engine Google was used with combinations of the search terms in Table 1. Lastly, the snowball strategy (pursuing references of references) was used to retrieve articles not located through the previous search strategies, which continued until January 2020.

### 2.2. Study Selection and Eligibility Criteria

The literature review was conducted by two researchers and was guided by the Preferred Reporting Items for Systematic Reviews and Meta-Analyses extension for Scoping Reviews (PRISMA-ScR) statement [40]. All types of documents were considered, including research articles, literature reviews, white papers, reports, directives, and National Documents. Documents from Europe and other occidental countries were taken into account, assuming cultural similarities. The titles and abstracts of documents identified through database, snowball and hand-searching were assessed against the inclusion and exclusion criteria defined in Table 2 and the relevance to the research question. Full text versions were downloaded to Refworks citation management software (ProQuest LLC, Ann Arbor, MI, USA, 2020), and then reviewed against the eligibility criteria prior to the final selection of documents for inclusion and revision. Likewise, the titles and executive summaries of grey literature were assessed against the inclusion and exclusion criteria before downloading the full text for review. Two independent researchers sequentially reviewed the titles, abstracts and full text, and any disagreements were resolved through discussion until consensus was achieved.

### 2.3. Data Charting

Two independent researchers entered the data into a data charting form using the program Microsoft Excel. The variables included for a standardized extraction of the data were: author, date published, country, type of document, research design, aim, setting and target population and relevant main findings. Any disagreements were resolved through discussion between the two researchers. Afterwards, the analysis of data was carried out by applying the analytical–synthetic approach. In the analytical process, the data were distributed and analyzed in additional parts, analyzing keywords and the content of the data. Then, in the synthetic process, the information and elements of the previous analysis were logically related to each other and integrated to make a meaningful contribution towards the development of the objective [41]. This led to a framework of the principle criteria that needed to be fulfilled in order to guarantee sustainable diet promotion within health services.

## 3. Results

**Figure 1:**
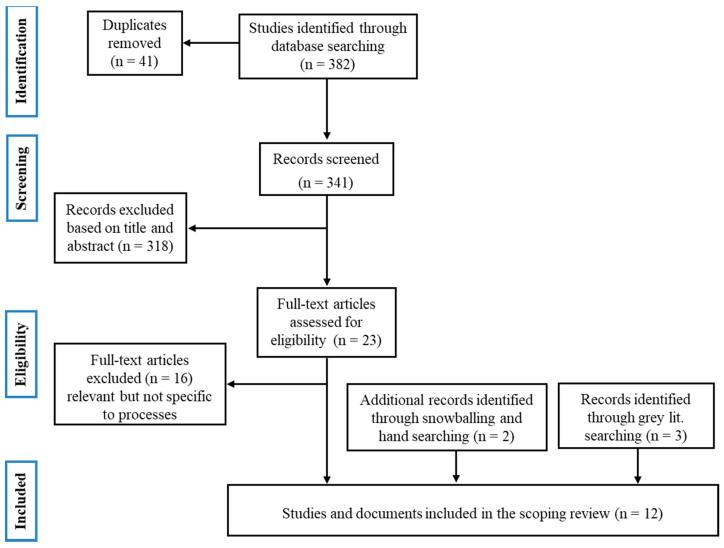
Preferred Reporting Items for Systematic Reviews and Meta-Analyses (PRISMA) flow diagram of included documents.

The initial database search yielded a total of 382 items, as shown in Figure 1, and 41 duplicates were removed. After screening for the titles and abstracts, 23 full-text articles were assessed for eligibility against the inclusion and exclusion criteria, as well as for relevance and specificity to the research question. From them, only seven articles were found to be relevant to the study. Three additional articles were found within the websites of relevant institutions, and finally, two articles were included that had been found by hand searching. In total, 12 full-text articles were included in the scoping review.

All of the retrieved articles were from 2007 onwards, with eight of them published in the last two years. Most of the articles came from the United States, Canada and the United Kingdom. Research articles (*n* = 5), research reviews (*n* = 2) and those associated with position papers, guides, and white papers (*n* = 5) were included. Table S1 contains the final article selection and a summary of the content analysis, including the main concepts, actions and approaches identified to work towards sustainable diet promotion in the healthcare system. Concepts such as governance, policy advocacy, education and literacy, leadership and community appear recurrently. However, a systemic vision perspective implies their interdependency, placing all the elements from the table at the same level of importance.

Six of the articles describe the necessity of including a system vision in the job responsibilities of health professionals in order to work towards sustainable food system advocacy [16,31,42,43,44,45]. In line with that system vision, articles describe the development of new roles of nutrition professionals, new responsibilities within a multilevel action approach necessary in order to advocate for sustainable food systems (SFSs) and to implement sustainable promotion within healthcare services [16,31,42,45,46,47].

Within those roles, organizational policy development and institutional involvement are present as key tasks to achieve in order to guarantee sustainable diet promotion and development and tackle inequalities [16,30,31,45]. This also includes the development of a food procurement policy within the healthcare organization as a tool for education, leading by example and leveraging sustainability [46].

A need for consensus on the definitions of a sustainable food system (SFS), diets and related concepts is highlighted in four articles, along with a need to develop standard operating procedures for practices that are focused on SFS and sustainable diet promotion [31,42,43,48].

Six out of the twelve articles focus on the need to improve and complete the higher education, training and professional development requirements towards sustainable food systems; this also includes the training of educators that are responsible for teaching the future nutrition professionals [16,30,31,42,43,47]. Many gaps are identified, for instance the low self-efficacy of dietitians due to little knowledge on sustainability matters [43,49]. Additionally, the articles suggest innovative changes in the curriculum content of nutrition-related health professionals in order to teach professionals from a system vision approach by including other areas not related directly to nutrition but with the food system (i.e., environmental sciences, economics or policy analysis and development). The need for practice training is highlighted, along with the lack of opportunity for it, as well as the need to push for continuous professional development (CPD) opportunities in the direction of sustainable food system education, emphasizing also the importance of peer-to-peer activity [47,48].

Social support and external leaderships are seen as strategic in order to maintain momentum towards sustainable means, along with a cultural transition from the current narrow view of dietetic practice [43,50].

**Figure 2:**
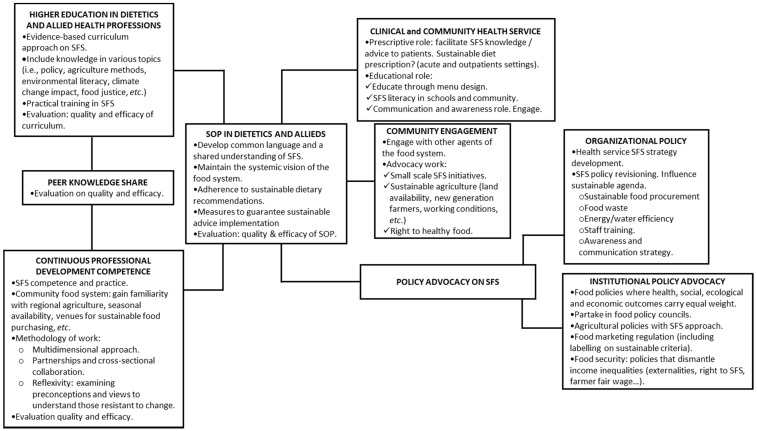
Theoretical framework visualizing the principal elements identified through the scoping review on sustainable diet promotion. Sustainable Food System (SFS).

Figure 2 shows the identified processes, circumstances and roles that need to be fulfilled in order to guarantee sustainable food promotion in healthcare systems. A central axis highlights the core importance of a standard operating procedure (SOP) based on a common understanding of the sustainable food system. From the central axis stem two focuses: the first on appropriate education and CPD to impact sustainability-based SOP implementation, and the second on the different key practices and the roles (both in the community and in public policy) necessary to pursue sustainable diet promotion through a common SOP.

## 4. Discussion

The very scarce literature on sustainable diet promotion in healthcare services illustrates that this is an emerging area of work. Studies still focus on defining the different responsibilities and roles that health professionals should carry out in order to leverage sustainable food systems within their practice. Most articles included in this review were published in the last two years and it is striking that the majority of this research has come out of the US and Canada.

The responsibilities of promoting sustainable diets within health services seems to still be reliant on the self-efficacy and individual beliefs of health professionals [49,50]. Hence, inclusion of sustainable food system parameters seems to be a personal choice rather than an agreed population-based promotion approach or protocol. In this sense, Worsley et al. (2014) showed that only a minority of nutrition professionals are interested in environmental sustainability and are receptive to further communication on the subject [51]. Furthermore, Hawkins et al. (2015) indicate that merely 34% agreed that registered dietitians should play a major role in climate change mitigation strategies [52].

This limited interest and lack of self-efficacy are linked to two areas of work yet to be developed. Firstly, the lack of knowledge, which leads to a lack of capacity to practice with a sustainable approach; and, in turn, it demonstrates a huge gap in the areas of higher education and CPD [30,31,42,43,47]. Secondly, within the profession, there is a need for consensus on the definition of a sustainable diet, supported by the introduction of related professional standards and protocols [30,31,42,43]. The importance of a clear and shared understanding of sustainable food systems in the healthcare system cannot be underestimated, as not having a shared framework and explicit and specific criteria undermines action [42]. The complexities surrounding the definitional aspects of sustainable diets and the lack of one clear definition hinders the translation of what a sustainable diet looks like on a plate, even for nutrition professionals [31,33].

Food system challenges have an inherent socioecological complexity that requires interdisciplinary approaches to problem solve [33]. The introduction of sustainability parameters within health professions implies new views on professional practice, roles and responsibilities. An example of the actions required are very well summarized by Bash and Donnelly with ten recommendations for public health professionals to support healthy and sustainable food systems, which involves not just prescriptive activities but also advocacy roles [44]. The Giessen Declaration highlighted this, stating that the “new nutrition science” needed to move beyond biomedical science to address ethical concerns that include social and ecological factors [53]. Hence, those who incorporate resilience and sustainability principles into practice often consider a food system approach [31,42,45,47]. This requires a multi-level and multi-criteria approach for nutrition professionals to ensure that the correct framework is in place for sustainable diet promotion.

While individual choice is a part of the equation, the food environment plays a significant role in how successful any change in individual behavior will be [54,55]. Dietitians employ behavior change strategies and empower individuals to make decisions to support a healthy and sustainable diet, but the food environment must support this positive decision-making [45]. Information-based approaches have shown limited efficacy unless used as part of a wider policy mix [24]. Citizens will only change consumption patterns when the food environment provides norms, opportunities and incentives to change behavior and facilitates an equitable access to affordable, sustainable and healthy food and the supporting infrastructures [23]. Health professionals should take part in the design of sustainable food environments within their communities [30].

### 4.1. Education

Pettinger (2018) stated that to translate complex, multi-disciplinary sustainability evidence and to influence the sustainability agenda, dietitians and nutritionists need to amplify their visibility, consolidate their skills and become more sustainability literate [31]. There is a need to evolve the training of professionals in order to pursue the implementation of sustainable healthy diet promotion from a multilevel approach. The scarce literature available is mostly focused on education and training, reflecting the need to effectively integrate a range of elements related to the leverage of sustainable food systems and sustainable diet promotion [16,30,31,42,43,47]. A new skillset is required by health professionals to work from a multidimensional vision of the food system [31,42,43] and to be part of stronger multi-sector leadership, in championing a sustainable ecological approach to the food system.

However, there is limited understanding and emphasis on the education and training needs within this emerging area of sustainability practice [43]. Moreover, educators for future nutrition and dietetics professionals have stated their lack of preparation for teaching sustainability matters [43,48]. Therefore, all the educative parameters in the teaching of nutrition students and active professionals need to be reviewed and adapted accordingly. Higher education, training and CPD need to step aside from the reductionist view of nutrition and health towards an integral food systems view [31,48]. Carlsson et al. [42] explained that international coordination is advisable to achieve an agreement from international and national dietetic/nutrition professional bodies, as food system sustainability literacy is an essential part of education, training and practice [56].

The curriculum needs to include new areas of knowledge that belong to the food system but are not exclusive to nutrition, such as environmental sciences, agriculture methodology, food system related policy, food system related strategy design, etc.; the cross-disciplinary approach within the curriculum is essential in order to empower professionals to include sustainability within their practice [31,47,48]. Innovative approaches have been suggested within an educational curriculum for dietetic and nutrition students to emphasize sustainability literacy, such as student-led sustainability-focused scenario-based approaches to learning that involve co-learning with students from other disciplines [31].

Practical training opportunities in sustainable food systems are scarce, and educators do not feel that they are well prepared to face this challenge [43]. Investing in student training and professional development that is grounded in a clear understanding of the terms, concepts and current issues is essential for practitioners to play a strategic role in the future [56].

CPD needs to prepare those that are currently active to guide the advocacy of sustainable food systems in the healthcare field [47,48]. Peer-to-peer education is a fundamental tool [48]. Additionally, identification of opportunities to engage in SFS advocacy-related work has been recognized as a powerful CPD tool. Opportunities for interdisciplinary communication and learning between dietitians and various stakeholders are essential, such as the hardly mentioned communication between sustainable agriculture farmers [47].

Therefore, meaningful ways to acquire up-to-date knowledge and skills to practice effectively within this emerging multidisciplinary field are needed, as well as evaluation methods for the professional capacity of healthcare workers [43,57].

### 4.2. Clinical and Community Health Service

Evidence suggests that the scope of practice in promoting sustainable food systems and the prescription of sustainable diets in healthcare services is still limited [43]. As an example, the study by Wilson and Garcia [49] included in this review, highlighted that dietitians advise a reduction in meat intake from a health perspective rather than an environmental one. This suggests that dietitians are practicing within a reductionist approach, instead of a system-thinking or integral approach [57]. One could argue that the lack of consensus or common language regarding a sustainable food system is the main cause, leading to a lack of SOPs for the implementation of sustainable diet promotion within the health service setting [42]. Even though international dietetics associations and other clusters, such as the EAT-Lancet Commission, have outlined the significance of implementing sustainable diets within their white papers and reports [30,45,57,58], the lack of SOPs, in addition to the gaps in knowledge of healthcare professionals, is a handicap for implementing sustainable diet promotion within the health services.

Nutrition health professionals should focus their community and clinical setting practices in guiding their clients and patients towards making ecologically sustainable food choices that are also healthy. For diet promotion to be feasible, health professionals need to make the sustainable food choice the easy choice for patients by facilitating the connection with local producers, exploring the venues where locally grown produce and animal products are sold directly to consumers and educating patients on seasonal foods and food citizenship (responsible consumption) [31,47].

In a clinical setting, one of the strongest options for advising and educating patients about sustainable foods is leading by example, with a sustainable food procurement strategy within the organization and menu planning that goes with it [16,44,59].

### 4.3. Community Engagement

Tagtow et al. said that the health benefits of sustainable food systems extend beyond single nutrients to create vibrant and resilient communities [16] by impacting directly on the social, economic and cultural dimensions of sustainability. Therefore, the multi-level approach to promote sustainable food systems infers working within a range of settings and with different agents of the food system, pulling away from the exclusive physiological/biological vision of nutrition into broader actions to address all the sustainable dimensions [43,44]. Nutrition professionals should include participation in community food projects; the development of connections between local farmers, producers, consumers and citizens; learning about seasonal foods; visiting farmers’ markets or food co-ops; and engaging in food policy discussions within their responsibilities [16,42,47]. They should advocate for local sustainable farmers, for access to land for new-entrant farmers and the fair price of produce, as well as other social issues such as easy access to sustainable and healthy food for all to avoid inequalities [42].

Community engagement has also been included as a learning resource for CPD; it gives nutrition professionals the information necessary to advise patients on how to access sustainable food within their communities [47].

### 4.4. Policy Advocacy—Governance

To catalyze the social paradigm change, various public policies affecting food systems should be urgently reformed to address climate change and biodiversity loss, to prevent obesity and non-communicable diseases and to make farming viable for the next generation [45]. Within multilevel and cross-sectorial activities, many of the studies included in this review address the need for dietitians and allied health professionals to work within the institutional and organizational level for policy advocacy (see Figure 2). New governance models to influence the sustainability agenda are required that include nutrition professionals to participate in local, regional and national legislative processes [16,30,31,42,45,47].

At the same time, at the organization level (such as in hospitals), nutrition professionals should involve themselves within a sustainable food system strategy development team. They should be involved in the design of the strategic processes to accomplish local and sustainable food consumption within the organization (i.e., public procurement, waste management, awareness and training) [16,42,47]. Hence, they must show some leadership skills and persuade the senior managers of the organization to move towards sustainable strategies rather than trading health for profit within the organization [16,49,59].

### 4.5. Social Support and Gender—Transversal Parameters

This review identifies two transversal conditions to consider within the role of nutrition professionals advocating for sustainable food systems.

Hawkins et al. [50] advise on the importance of social support, including networking with other supportive partnerships, as a crucial factor in sustaining pro-environmental behaviors. One important partnership which is hardly covered in current literature is the collaboration with farmers. Relationships with agents of the food system are pivotal to keep motivation high and, in the case of farmers, it helps individuals to reconnect with the land and the food we consume. The lack of mention of farmers in the literature is proof of the lack of a systemic vision and process approach. External professional peers are also seen as important partners in the transition towards sustainability, such as the Sustainable Food System Leadership Team from the Dietitians of Canada that provide guidance on relevant issues related to sustainable food systems in their health system [43]. In these support networks, in addition to farmers and external peers, managerial support within the health organization has been shown to be influential in the wider incorporation of environmental issues [43].

The second transversal concept is the gender approach. The studies included in this review showed that women are prominently represented in nutrition-related professions. The historic split of working roles between genders, where the “feeding” side has always been considered the women’s role, is still present [59]. Although the question of gender has not been a central axis of analysis in this review, care must be taken as feminized jobs are normally “hierarchically” minor, invisible or less valued, as current vertical structures are based on masculinized logic [60]. Therefore, it is fundamental to take a feminist perspective into account to avoid the continued invisibilities that hinder the public and political work and to build fairer environments for all professionals in the development and advocacy of sustainable food systems.

### 4.6. Limitations

Despite the methodological care taken with this research and the reliability of the sources analyzed, a limitation of this scoping review comes from the small sample size of papers (i.e., in the scope of the articles related to sustainability improvement of the diets of patients who leave the hospital). We consider that this has come about because of the novelty of the research question regarding sustainable diet promotion processes in healthcare systems, which confirms the need for the objective of this research and the methodology used and opens up new and important research lines.

## 5. Conclusions

The healthcare system has a unique opportunity to protect environmental and public health by implementing sustainable food practices in its institutions. Evidence gathered in this review article suggests that nutrition-related health professionals have a huge potential to leverage change; however, currently, their role and impact level is underestimated by healthcare systems in clinical as well as in community settings. Sustainable diet promotion in healthcare services is not yet fully nor equally implemented, as it is more driven by personal beliefs or implications than by actual SOPs that set specific protocols for practice.

This review has identified a framework with key areas of work in order to guarantee sustainable diet promotion in healthcare services (Figure 2). Health professionals need to take on board emerging new roles and responsibilities, including policy advocacy and community engagement. Innovations in the education curriculum and training on sustainability matters for these professionals, along with political and social empowerment work, are required. For that, there is a need to develop a common language and a shared vision in sustainable food systems and sustainable diet promotion, with instruments to assess the degree of implementation and impact.

All of the above require a process approach for transforming the current reality to a desired one by enabling the participation of key stakeholders that come from different realities (i.e., farmers). The process´ leadership needs to embrace a systemic view of the food system, with a rightful distribution of the value and importance of the stakeholders.

The challenges shown in this review also feed into the areas of research and innovation that need to be developed within the healthcare system in order to enable the promotion of sustainable diets within healthcare services.

The time is ripe for health professionals to cultivate a systems approach which optimizes the nutrition and health of all eaters while preserving the environment and re-localizing the economy. Also, from a feminist perspective, it is essential to question the leadership model and the established roles and determine time and resources to promote inclusiveness and the mobilization of said roles and privileges.

For the change in the social paradigm towards consolidating sustainable food systems, it is essential to define the role of health professionals and the health system as key for the promotion of sustainable diets.

## Figures and Tables

**Table 1:** Search terms used for each of the concepts.

Concept	Search Terms
Health services	(“Health services” OR “health system” OR “healthcare” OR Hospitals OR “health professionals” OR dietitian OR nutrition professionals OR “community nutrition” OR “Public Health”) *AND*
Sustainable diet	(“sustainable food” OR “sustainable diet*” OR “sustainable eating” OR sustainab* nutrition OR “planetary health diet” or “organic food”) *AND*
Process	(Policy* OR guideline* OR regulati* OR sustainable guidelines OR treatment OR therapy OR promotion OR counsel?ing OR education OR advice OR prescription)

(*) The asteriks is a truncation command that instructs the database to search for the root of the word typed in and then retrieve any alternate endings.

**Table 2:** Inclusion and exclusion criteria.

Concept	Inclusion Criteria	Exclusion Criteria
Language	English	All other languages
Year	>2000	<1999
Country	European and international studies with relevance to Europe	International studies without relevance to Europe
Setting	Healthcare system and public settings	Non-healthcare systems
Type of document	No restrictions	

## Data Availability

The data presented in this study are available in Supplementary Material.

## Supplementary Materials

The following are available online at https://www.mdpi.com/2072-6643/13/3/747/s1, Table S1: Summary of content analysis of the retrieved articles.
