# Supplementary material for: The Promotion of Sustainable Diets in the Healthcare System and Implications for Health Professionals: A Scoping Review
Source: Nutrients. 2021 Feb 26;13(3):747. doi: 10.3390/nu13030747 (PMC7996772; doi:10.3390/nu13030747)
Supplement: Supplementary file 1 [file nutrients-13-00747-s001.zip › nutrients-1110471-SI/Table S1.pdf]

Table S1. Summary of content analysis of articles retrieved. Sustainable food systems (SFSs); Continuous Professional Development (CPD).

Author (year) Country	Type of document	Content analysis of main findings
Carlsson, *et al* (2019) (42) Canada	Research article	- SFS **vision** must be multidimensional and multilevel.<br>- Sustainable policy **frameworks** are required.<br>- **Governance** structures that work against current power imbalances and inequality within the food systems are required.<br>- **Consensus** on SFS required among health professionals (the dietetic profession) for use of common language and understanding.<br>- Sustainability **literacy** for nutrition professionals: education and professional development opportunities.<br>- Support of **education** on SFS in schools and community<br>- Institutional and organizational **policy advocacy** on SFS, giving equal weight in advocacy messages of health, social, economic and ecological outcomes, to regulate food system.<br>- **Action** approaches: cross-sectorial networks and reflexive approaches.<br>- **Evaluation** tools to track progress towards the vision.
Champ CE, *et al* (2019) (46) United States	Research article	- **Governance** structures: stimulating and prioritizing the decision making power of the organization, not the industry.<br>- Organizational **Policy advocacy** on SFS: transition from "for profit" to "leading by example" vision.<br>- **Action** approaches: l**ong-term approach** for supply and education strategies.
Wegener J (2018) (43) Canada	Research article	- Sustainability **literacy** for nutrition professionals: education, training and professional development on SFSs (also directed to educators, as 68% felt inadequately prepared). Incorporate environmental topics into public health nutrition and dietetics practice. Current training opportunities are limited.<br>- Professional **self-efficacy** development: knowledge-enhancing opportunities and experiential knowledge to adopt SFS in practice.<br>- Standard of practice (**SOP**) needs to include promotion of SFS and dietary practice.<br>- **Leadership**: Supporting network of professionals.<br>- **Multidisciplinary** area of work and a clear view of the multiple benefits of SFS activity attainable through professional public health action.<br>- **Conflicts of interest**: the integrity of industry-funded research and resources in education and training evaluated.

Table S1. (*cont.*) Summary of content analysis of articles retrieved. Sustainable food systems (SFSs); Continuous Professional Development (CPD).

Author (year) Country	Type of document	Content analysis of main findings
Pettinger C (2018) (31)<br><br>United Kingdom	Review article	- SFS **vision**: needs a system-thinking approach to problem solving in food systems.<br>- **Consensus** on sustainable diet required: professional standards would support consensus development.<br>- Sustainability **literacy** for nutrition professionals: a need to 'amplify their visibility', consolidate their skills and become sustainability literate. Integration of "environmental literacy" in undergraduate nutrition programs.<br>- **Curricula** innovations, including other disciplines.<br>- Institutional and organizational **policy advocacy** on SFS: nutrition professionals should have the skillset to be part of ongoing high-level policy discussions in SFSs area and to influence local, regional, national policy and the sustainability agenda.<br>- **Multidisciplinary** area. Commitment for change. Nutrition professionals have an important role supporting people to become food citizens and to advocate for democratic and SFSs.
Wegener *et al*. (2018) (48)<br><br>Canada	Review article	- Sustainability **literacy** for nutrition professionals (including educators): university, practical training and CPD (peer-to-peer).<br>- **Curricula** innovations, including other disciplines. **Training** tools: specifically critical reflection and experiential learning.<br>- Professional **self-efficacy** development: Currently there is a lack of implementation of SFS concepts in dietetic practice due to a lack of knowledge and personal perspective among educators.<br>- **SOP**: integrate food sustainability in dietetic performance standards, professional competencies and development.
Hawkins I W, *et al*. (2015) (50)<br><br>United States	Research article	- **Networking**: social support, including partnerships, is considered a crucial factor in sustaining pro-environmental behaviours.<br>- Professional **self-efficacy** development: The role of fulfilment helps and a sense of personal gratification with regard to diet-related efforts to mitigate climate change encourages personal commitment.<br>- **SOP**: the lack of SOP leads to a correlation between personal belief and professional behaviour.
Wilson ED & Garcia AC (2011) (49)<br><br>Canada	Research article	- **Gender**: 87% were female.<br>- Sustainability **literacy** for nutrition professionals. Perceived lack of knowledge in sustainability parameters of the food system.<br>- **SOP**: Personal beliefs instead of setting SOPs within practice. Currently, environmental impact of food is not central to practice.<br>- **Leadership**: Dietetic professionals, as food experts, can potentially lead the way in education and policy and practice changes. Also gaining support from senior leaders is deemed as important.

Table S1. (*cont.*) Summary of content analysis of articles retrieved. Sustainable food systems (SFSs); Continuous Professional Development (CPD).

Author (year) Country	Type of document	Content analysis of main findings
EFAD (2019) (45)<br><br>Europe	White paper	- **SFS vision**: actions must be based on the systemic view of sustainability.<br>- **Networking**: link with organisations locally, nationally and throughout Europe to promote sustainable food production while promoting healthy body weight.<br>- Institutional and organizational **policy advocacy** on SFS.<br>- Action **approaches**: multilevel action of dietitians to obtain population change.<br>- In Europe, **five paradigm shifts** to build SFSs: 1. Ensure access to land, water and healthy soils; 2. Rebuild climate-resilient, healthy agro-ecosystems; 3. Promote sufficient, safe, healthy, sustainable diets; 4. Build fairer, shorter and cleaner supply chains; 5. Put trade in the services of sustainable development.
Harmon A H, *et al*. (2007) (47)<br><br>United States	Position paper	- Sustainability **literacy** for nutrition professionals. Gain familiarity with regional agriculture and seasonal availability, and then explore the venues where locally grown produce and animal products are sold directly to consumers. Knowledge on environmental concerns should be increased through continued professional development and research.<br>- Support of **community** education on SFS. Role of dietetic practice in supporting community SFSs. Equipping community with knowledge on sustainable diet content and local food sources.<br>- Institutional and organizational **policy advocacy** on SFS. Participation in legislative processes.<br>- Action **approaches**: variety of roles and responsibilities of nutrition professionals in sustainable actions development.
BDA (2019) (30)<br><br>United Kingdom	Document guide	- **Consensus** on SFS: understanding sustainable diets and moving towards a consensus on definition.<br>- Sustainability **literacy** for nutrition professionals: at university, training, and CPD level.<br>- Support of **community** education on SFS: school curriculum and community education. Ensure a clear and relevant message for all, accommodating different cultures. Helping consumers to make food choices for sustainable diet.<br>- Institutional and organizational **policy advocacy** on SFS: multi-organisation involvement: Improve the availability of sustainable foods by developing integrated multi-organisation sustainable polices with government, local authorities, farmers, local producers, NGOs and commercial companies.<br>- **Leadership**: dietetic professionals as leaders on food behaviour and environment and health.

Table S1. (*cont.*) Summary of content analysis of articles retrieved. Sustainable food systems (SFSs); Continuous Professional Development (CPD).

Author (year) Country	Type of document	Content analysis of main findings
Tagtow A & Harmon A (2009) (16)<br><br>United States	Document guide	- Sustainability **literacy** for nutrition professionals. Incorporate SFS concepts into university courses, internships, and research agendas.<br>- Support **community** education on SFS. Connection with community´s food system: providing consumers with information to influence the food choices, behaviours, and engaging in local and global food system issues. Contribute to stronger local food systems, and connect local producers with consumers.<br>- Institutional and organizational **policy advocacy** on SFS within in all sectors of the food system.<br>- Action **approaches**: the variety of roles of nutrition experts evokes the system vision of sustainability. Including sustainably produced food procurement as well as energy and water conservation and waste minimization strategies in the institutions.<br>- **Leadership**: food and nutrition experts have the opportunity to be a critical element in the foundation of an SFS.
Bash & Donnelly (2019) (44)<br><br>United Kingdom	Discussion paper	- Action **approaches**: ten multi-level and cross-sectorial actions that involve a wide range of roles that public health professionals can embrace to support SFS, which shows the systemic view requirements of the SFS advocacy and support:<br>1. Promote diets that prioritise plant-based proteins and a 'less and better' approach to animal-based foods;<br>2. Advocate for agriculture policy that considers a systems approach to agriculture and human health across the socioeconomic gradient;<br>3. Advocate for all agricultural trade agreements to support public health and environmental sustainability;<br>4. Advocate for reduction in antibiotic use in the livestock sector;<br>5. Take a global view on food systems and align public health policies with key international agreements (i.e. SDGs, Paris Agreement);<br>6. Support regulation for labelling related to food production methods;<br>7. Stimulate demand for sustainable food including local vegetables, pulses and fruits through public procurement;<br>8. Commission food programmes that support/promote SFSs;<br>9. Support community-based agriculture schemes that bring farming and green spaces into the urban and periurban environments of the local community.<br>10. Develop and support local policies and contracts that aim to reduce food waste within public sector food provision and large-scale catering.
